# Factors associated with the safety culture of patients under dialysis in the context of the COVID-19 pandemic

**DOI:** 10.1590/0034-7167-2022-0280

**Published:** 2023-02-03

**Authors:** Tatiana Aparecida Rodrigues, Fabrícia Moreira Amorim Amaral, Marília Alves Hoffmann, Cissa Azevedo, Helen Cristiny Teodoro Couto Ribeiro, Luciana Regina Ferreira da Mata

**Affiliations:** I Universidade Federal de São João del-Rei. Divinópolis, Minas Gerais, Brazil; II Universidade Federal de Minas Gerais. Belo Horizonte, Minas Gerais, Brazil

**Keywords:** Patient Safety, Safety Management, Organizational Culture, Dialysis, COVID-19., Seguridad del Paciente, Gestión de la Seguridad, Cultura Organizacional, Diálisis, COVID-19., Segurança do Paciente, Gestão da Segurança, Cultura Organizacional, Diálise, COVID-19.

## Abstract

**Objectives:**

to assess the factors associated with the safety culture of patients under dialysis in the context of the COVID-19 pandemic.

**Methods:**

a cross-sectional and analytical study, carried out in Minas Gerais, with 134 professionals from three dialysis services. The Hospital Survey on Patient Safety Culture, adapted for Brazil, was used.

**Results:**

only variable type of management was associated with the highest percentage of positive response in public and private services. Patient safety was rated as good by 55.7% of respondents. In dimension assessment, the public service presented one strength and five weaknesses, the private service did not present weak areas, and the philanthropic service presented a weakness. The priority areas for improvement actions are represented by dimensions “Nonpunitive response to error” and “Staffing”.

**Conclusions:**

interventions should consider the type of service management, as it is a factor associated with safety culture.

## RESUMO

**Objetivos:**

avaliar os fatores associados à cultura de segurança do paciente em diálise no contexto da pandemia de COVID-19.

**Métodos:**

estudo transversal e analítico, realizado em Minas Gerais, com 134 profissionais de três serviços de diálise. Utilizou-se o instrumento *Hospital Survey on Patient Safety Culture*, adaptado para o Brasil.

**Resultados:**

apenas a variável tipo de gestão esteve associada à maior porcentagem de resposta positiva nos serviços púbicos e privados. A segurança do paciente foi classificada como boa para 55,7% dos respondentes. Na avaliação das dimensões, o serviço público apresentou uma fortaleza e cinco fragilidades, o serviço privado não apresentou áreas frágeis, e o serviço filantrópico apresentou uma fragilidade. As áreas prioritárias para ações de melhoria estão representadas pelas dimensões “Resposta não punitiva para erros” e “Dimensionamento de pessoal”.

**Conclusões:**

intervenções devem considerar o tipo de gestão do serviço, por ser um fator associado à cultura de segurança.

## RESUMEN

**Objetivos:**

evaluar los factores asociados a la cultura de seguridad del paciente en diálisis en el contexto de la pandemia de COVID-19.

**Métodos:**

estudio transversal y analítico, realizado en Minas Gerais, con 134 profesionales de tres servicios de diálisis. Se utilizó el instrumento *Hospital Survey on Patient Safety Culture*, adaptado para Brasil.

**Resultados:**

solo la variable tipo de gestión se asoció con el mayor porcentaje de respuesta positiva en los servicios públicos y privados. La seguridad del paciente fue calificada como buena por el 55,7% de los encuestados. En la evaluación de las dimensiones, el servicio público presentó una fortaleza y cinco debilidades, el servicio privado no presentó áreas frágiles y el servicio filantrópico presentó fragilidad. Las áreas prioritarias de acciones de mejora están representadas por las dimensiones “Respuesta no punitiva a los errores” y “Escalamiento de personal”.

**Conclusiones:**

las intervenciones deben considerar el tipo de gestión del servicio, ya que es un factor asociado a la cultura de seguridad.

## INTRODUCTION

Renal impairment in hospitalized patients with COVID-19 infection associated with increased hospital mortality and the worst clinical evolution of these patients implies the need for strategies to promote patient safety in dialysis services^(1)^.

It is noticeable that new challenges have emerged in dialysis centers in the face of the outbreak of the COVID-19 pandemic, demanding a reorganization of services for a safe clinical practice for chronic renal patients under dialysis^(2)^. The creation and validity of protocols with the flow of care for screening suspected or confirmed patients with COVID-19 as well as the elaboration of a management care plan, were primary strategies adopted by some services^(2)^. Others focused their actions on the implementation of preventive measures and the use of telehealth as a remote monitoring tool for patients^(3)^. However, no studies were found in the literature to assess safety culture in hemodialysis services in the context of the COVID-19 pandemic, considering the different types of management.

COVID-19 disease manifests itself mainly as a respiratory disease of mild symptoms, similar to those of influenza^(4)^. However, depending on the risk factors, not yet fully understood, acute respiratory syndrome may occur and result in the development of acute kidney injury^(4)^. In such cases, renal replacement therapy may be necessary, which impacts the overload of dialysis services, which are considered complex sectors.

The clinical profile of patients involving individuals in severe clinical conditions with comorbidities may increase the chances of adverse events occurring^(5)^. Moreover, in Brazil, the number of patients with end-stage renal disease has been increasing at a faster rate than the planned infrastructure expansion of dialysis facilities, which exceeds the national capacity to manage the system. Therefore, the increase in the number of patients dependent on renal replacement therapy as a result of COVID-19 implies serious consequences in the care and management scope, which can compromise patient safety and quality of care^(6)^.

In recent years, patient safety has been discussed with concern regarding the impact of adverse events in the context of hemodialysis^(7)^. The advent of the COVID-19 pandemic has increased the need for discussions on patient safety culture. This is because the overcrowding of health services and the work overload among professionals favor the increase in the occurrence of adverse events and, consequently, the risks of morbidity and mortality for the population^(8)^.

In 2013, the Brazilian Ministry of Health instituted the National Patient Safety Program^(9)^, which proposes the promotion of a safety culture with an emphasis on learning, organizational improvement, the engagement of professionals and patients and the prevention of incidents. Safety culture, in turn, is defined as the product of individual and group values, attitudes, perceptions, competencies and behavior patterns that determine commitment, style and proficiency with the health and safety management of an organization^(10)^.

To establish an effective safety culture, periodic assessment is essential that is able to trace local diagnosis in order to direct intervention planning and improvement monitoring^(11)^. Among the tools used to measure patient safety culture, the Hospital Survey on Patient Safety Culture (HSOPSC), developed by Agency for Healthcare Research and Quality (AHRQ)^(12)^, stands out. This is a self-administered questionnaire that assesses the weaknesses and strengths of safety culture, allowing reflections for the elaboration of intervention measures, with a view to improving quality of care^(13)^.

In the contexts of Brazilian nephrological practices, there are still gaps in knowledge about the different levels of patient safety culture in dialysis services, especially in the context of the COVID-19 pandemic. Culture assessment, from the perspective of its determinants in different types of management, points to possible improvement interventions that boost quality of care and patient safety^(14)^. Assessing the safety culture is also a fundamental activity to promote it, as it builds a baseline and enables monitoring of the effects of internal interventions and improvement policies^(15)^. Furthermore, hazards inherent in high exposure during the COVID-19 pandemic, associated with the greater physical and mental exhaustion experienced by health professionals, enhance the importance of assessment in the present moment^(16)^. Therefore, the following question emerged: how is patient safety culture configured and what are the associated factors in three dialysis services in the context of the COVID-19 pandemic?

## OBJECTIVES

To assess the factors associated with the safety culture of patients under dialysis in the context of the COVID-19 pandemic.

## METHODS

### Ethical aspects

The present study was approved by the Research Ethics Committee of the proposing institution and the other institutions that were included as co-participants, seeking to meet the ethical precepts for research, according to the Resolution of the Brazilian National Health Council 466/2012.

### Study design, period, and location

This is a cross-sectional and analytical study, described from the STrengthening the Reporting of OBservational studies in Epidemiology (STROBE) recommendations^(17)^. Data collection was performed in three dialysis services in the state of Minas Gerais, Brazil, with different types of management: public, philanthropic and private. The project was developed from March to June 2021. This period was considered the peak of the COVID-19 pandemic in Brazil, due to the higher lethality from the disease, according to data from the Ministry of Health’s Epidemiological Bulletin^(18)^.

### Population: sample definition and inclusion and exclusion criteria

The selection of participating services was based on the choice of different administrative nature, which makes it possible to broaden the discussions on the influence of determinant aspects of safety culture, such as the complexity, particularities and service structure in each organization, in different administrative scenarios.

The public dialysis service is inserted in a federal university and general hospital, a reference in the municipal and state health system in medium and high complexity care. It performs about 1,000 hemodialysis sessions monthly. The philanthropic dialysis service is located in an academic hospital, which allocates 100% of its care to patients in the public health system of the capital, metropolitan region and countryside, referred by the Municipal Health Department, with an average care of 900 hemodialysis sessions per month. The private dialysis service is a for-profit institution, located in a satellite clinic, which is not part of a hospital and has the capacity to care for up to 400 hemodialysis sessions per month.

All 134 professionals from the multidisciplinary and administrative health team who worked in the three dialysis services were invited to participate in the study. According to the AHRQ recommendations, for studies involving services with fewer than 500 professionals, the entire team should be considered for sample composition^(12)^.

Thus, we considered medical professionals, the nursing team (nurse, technician and nursing assistant), multidisciplinary assistance (social worker, nutritionist, psychologist and pharmacist) and the administrative sector. Of the 134 professionals invited, 72 were from the pubic dialysis service, 33 from the philanthropic dialysis service and 29 from the private dialysis service. We included professionals from the multidisciplinary team of dialysis services regardless of institutional affiliation and working for at least three months at the study site. We excluded professionals with no employment relationship with the services (interns, residents, preceptors and academics), professionals absent due to vacation, health leave, pregnancy leave and/or medical certificate and questionnaires that presented less than 50% of completed answers were excluded.

### Study protocol

The instrument used for collection was the HSOPSC questionnaire, electronic version, adapted to the Brazilian reality, plus some opportunities for improvement. It consists of 42 questions, which make up 12 the safety culture dimensions: D1. Frequency of event reporting (3 items); D2. Perception of safety (4 items); D3. Supervisor/manager expectations & actions promoting safety (4 items); D4. Organizational learning-continuous improvement (3 items); D5. Teamwork within units (4 items); D6. Communication openness (3 items); D7. Feedback and communication about error (3 items); D8. Nonpunitive response to error (3 items); D9. Staffing (4 items); D10. Hospital management support for patient safety (3 items); D11. Teamwork within units (4 items); e D12. Hospital handoffs & transitions (4 items).

The 42 questions related to patient safety culture were constructed based on a Likert scale of five alternatives: totally disagree; disagree; neither agree nor disagree; agree; totally agree; or never; almost never; sometimes; often; and always. The questionnaire also includes a question of global qualification of patient safety level, obtained through the global safety score, which varies from 1 to 10 (1 and 2, poor; 3 and 4, poor; 5 and 6, fair; 7 and 8, good; and 9 and 10, excellent) and a question about the number of safety incidents reported in the last year.

The instrument in electronic version, entitled “*E-Questionário de Cultura de Segurança Hospitalar*”, was developed, translated, adapted and validated by the *QualiSaúde* research group, *Universidade Federal do Rio Grande do Norte*, applied in partnership with the Brazilian National Health Regulatory Agency (*Agência Nacional de Vigilância Sanitária*). Validity was verified by confirmatory factor analysis and reliability by consistency analysis with Cronbach’s alpha calculation^(12-13)^.

As it is a standardized questionnaire, known worldwide and adapted to the Brazilian context, the authors did not adapt the instrument to perform data collection. The focus was to present the results and discussions considering the moment and the reality in which the data were collected, perpetuated by the context of the COVID-19 pandemic, which can impact the perception and responses to the instrument items.

Initially, the participating services’ immediate supervisors were asked to fill in a list with professionals’ electronic addresses, in order to send the questionnaire, and the WhatsApp contact, for quick communication in cases of need. Before starting the data collection process, the survey was disseminated to professionals of each service, through reminders via WhatsApp, prior to each sending of the survey by e-mail. In order to obtain as many responses as possible, three attempts were made for each professional, via e-mail, within seven, 10 and 15 days after the first submission of the questionnaire. Subsequently, to increase the percentage of responses and allow the participation of professionals who did not access the e-mail, data collection was complemented in the face-to-face mode, by two previously trained researchers, in different shifts, from questionnaire self-completion on tablets made available by the researchers.

### Analysis of results, and statistics

The Statistical Package for the Social Sciences, version 22.0, was used for data analysis. First, a descriptive analysis was performed for participant characterization. Data normality was verified using the Shapiro-Wilk Test, in order to guide the conduction of the types of tests used.

Inferential analysis was developed to compare the patient safety assessment score between services, the HSOPSC’s percentage of positive responses and its dimensions, and to assess the modeling of factors associated with the percentage of positive responses of each service. To compare the classification of patient safety scores in the services, the chi-square test of independence was developed, using the likelihood ratio as the critical ratio, due to the existence of an expected count lower than five in more than 20% of cells of 3x3 contingency table prepared.

The percentages of positive responses were presented in each safety culture dimension and in the total dimensions, as well as their respective 95% confidence intervals (95%CI), according to the type of service and professional training. The safety culture dimensions were classified in strengths when the items presented 75% or more of positive responses, and as weaknesses, when the percentages of positive responses were equal to or less than 50%^(12)^.

To construct the explanatory model, considering as an outcome the patient safety note, the generalized linear model application was considered by means of generalizable estimation equations (GEE), which allows analysis when there is a suspicion of violation of independence of data from participants in a clustering situation, as occurs in health units/services^(15-16)^. Therefore, in GEE, we considered the variable type of management as subject/cluster, and that of participants, as within the subject/cluster. Additionally, the unstructured work correlation matrix, an identity binding function and the log-linear distribution were used, since the outcome refers to a continuous metric.

To test the relevance of each independent variable in the model, the Wald chi-square test and fit quality by Quasi-likelihood under Independence Model Criterion interpretation were applied to GEE. As a measure of effect, the equation coefficient (β) was analyzed, which, in positive values, indicate a directly proportional association, and in negative values, an inversion relationship with the outcome. A significance level of 5% was adopted to minimize a type I error^(19-20)^.

## RESULTS

The “*E-Questionário de Cultura de Segurança Hospitalar*” was referred to 134 professionals, of whom 115 answered (86%). Of the answered questionnaires, one was excluded for presenting less than 50% of the answers. Thus, the sample was composed of 114 professionals, who presented a mean percentage of valid responses of 92.0% (95% CI = 93.9-90.1). Participant sociodemographic characteristics are described in Table 1.

**Table 1:** Characterization of professionals participating in the three dialysis services, Belo Horizonte, Minas Gerais, Brazil, 2021

	General		Public service		Philanthropic service		Private service	
	**n**	**%**	**n**	**%**	**n**	**%**	**n**	**%**
Response rate	114	92.03	65	90.3	21	75.9	28	84.8
Profession								
Administration	9	7.9	2	3.1	2	7.1	5	23.8
Multidisciplinary	9	7.9	6	9.2	1	3.6	2	9.5
Physicians	29	25.4	16	24.6	8	28.6	5	23.8
Nursing	56	49.1	36	55.4	14	50.0	6	28.6
Missing	11	9.6	5	7.7	3	10.7	3	14.3
	**M**	**SD**	**M**	**SD**	**M**	**SD**	**M**	**SD**
Working time in service	6.99	±5.55	8.87	±5.91	6.12	±3.70	2.00	±1.82
Working time at the unit	7.16	±5.68	7.1	±4.81	7.68	±6.05	6.42	±7.76
Hours weekly	30.22	±13.31	31.69	±9.91	27.32	±13.95	29.26	±16.50
Number of adverse event reporting	1.60	±3.23	1.72	±2.56	1.80	±5.11	0.94	±1.58
Specialization time	12.89	±7.72	14.58	±6.83	10.32	±7.80	10.95	±9.19

*N - simple frequency; M - mean; SD - standard deviation.*

In the assessment of patient safety attributed by employees of participating services, in general, 55.7% (59) of professionals of the three services rated patient safety as good (grade 7 or 8), and there was no classifying assessment for the poor category. It can be highlighted that, among the three institutions, the philanthropic service had the highest proportion of excellent grades among its employees (40.0%), followed by the private service (36.8%) and the public service (17.7%). Moreover, only in the public service there was a poor rating (4.8%) among its professional staff. However, there is no distinct distribution of notes between services (x^2^ = 10.29; p = 0.113).

In the analysis of the three services together, a percentage of 62.3% was identified for the percentage of positive responses, which refers to the general perception for all safety culture dimensions. The percentage of positive responses was higher in “D4” and “D3”, with 85.6% and 76.6%. “D8” and “D9” were classified as weak by the general sample, with a percentage of positive responses of 29.3% and 49.3%, respectively (Table 2).

**Table 2:** Comparison of patient safety culture dimensions between dialysis services, Belo Horizonte, Minas Gerais, Brazil, 2021

Dimensions	General	Public service	Philanthropic service	Private service
	PPR% (95%CI)^*^	PPR% (95%CI)^*^	PPR% (95%CI)^*^	PPR% (95%CI)^*^
Percentage of positive responses	62.3^†^ (67.2-57.4)	56.0^†^ (61.7-50.3)	68.3^†^ (73.8-62.8)	72.1^†^ (77.5-66.7)
D1 - Frequency of event reporting	58.9^†^ (63.4-54.5)	48.2^‡^ (56.2-40.1)	73.2^†^ (81.4-65.1)	71.7^†^ (79.6-63.8)
D2 - Perception of safety	53.9^†^ (84.6-23.2)	49.2^‡^ (82.4-16.1)	58.3^†^ (87.1-29.5)	62.8^†^ (90.2-35.5)
D3 - Supervisor/manager expectations & actions promoting safety	76.60^§^ (83.4-69.8)	71.5^†^ (83.5-59.5)	84.5^§^ (89.9-79.0)	81.7^§^ (83.9-79.5)
D4 - Organizational learning-continuous improvement	85.6^§^ (95.8-75.5)	80.0^§^ (93.8-66.1)	91.6^§^ (101.0-82.3)	95.2^§^ (95.2-95.2)
D5 - Teamwork within units	72.8^†^ (77.9-67.8)	69.6^†^ (74.8-64.3)	75.6^§^ (81.7-69.6)	79.4^§^ (87.8-70.9)
D6 - Communication openness	56.0^†^ (75.5-36.6)	48.7^‡^ (71.9-25.5)	69.9^†^ (85.2-54.6)	61.0^†^ (78.7-43.3)
D7 - Feedback and communication about error	60.1^†^ (65.5-54.6)	53.5^†^ (58.7-48.4)	68.6^†^ (80.1-57.1)	69.3^†^ (77.4-61.2)
D8 - Nonpunitive response to error	29.3^‡^ (47.4-11.2)	19.0^‡^ (37.0-1.0)	37.3^‡^ (62.5-12.1)	52.2^†^ (67.4-36.9)
D9 - Staffing	49.3^‡^ (70.3-28.3)	41.8^‡^ (69.2-14.5)	64.7^†^ (78.3-51.1)	52.1^†^ (66.4-37.8)
D10 - Hospital management support for patient safety	63.5^†^ (69.7-57.4)	53.6^†^ (57.7-49.6)	77.6^§^ (95.4-59.9)	75.7^§^ (80.0-71.4)
D11 - Teamwork within units	55.9^†^ (65.2-46.7)	46.5^†^ (54.4-38.6)	67.7^†^ (74.3-61.1)	75.8^§^ (94.8-94.8)
D12 - Hospital handoffs & transitions	56.7^†^ (66.2-47.2)	52.7^†^ (62.2-43.1)	60.0^†^ (76.6-43.5)	68.9^†^ (73.4-64.4)

PPR - Percentage of Positive Responses; ^^*^^95%CI - 95% Confidence Interval;

† Dimensions classified as opportunities for improvement;

‡ Dimensions classified as weaknesses;

§ Dimensions classified as strengths.

Both the general sample and each service analysis did not present a percentage of positive responses above 75%. However, it is possible to affirm that the best performance was private service, with 72.1% (77.5-66.7), which is significantly different from the philanthropic service and the public. The latter also had a significantly different proportion of positive responses (Table 2).

When considering separately the dimensions assessed in each service, it is clear that the private service was the one that presented the highest number of dimensions classified as strengths (D3, D4, D5, D10 and D11), followed by the philanthropic service (D3, D4, D5, D10) and the public service (D4). Regarding the dimensions assessed with a low percentage of positive responses, the public service was the one with the highest levels of weakness (five dimensions), followed by the philanthropic service (one dimension). Weaknesses were not observed in the private service (Table 2).

When analyzing the professional categories, it is evident a general pattern observed in the services, in which the profile of positive responses was, for the most part, between the values 50 and 75%, being classified as situations of opportunity for improvement (Table 3). However, in the culture dimension assessments, it is possible to see distinctions, such as the existence of three weaknesses for the nursing team (D1, D8, D9) and multidisciplinary (D2, D8, D9), two weaknesses for the administrative team (D8, D9) and a weakness in the medical team (D8). Regarding strengths, it is observed that there is only one reported by nursing (D4), three by the medical and multidisciplinary team (D3, D4, D5) and four by the administrative team (D1, D3, D4, D7). A wide 95%CI was observed in two dimensions (D2, D3), which demonstrates a great variability of views within the professional categories regarding the components of each dimension, making them statistically similar. As in service analysis, “D8” and “D9” present weaknesses for the professional teams, except “D9” for the medical team (Table 3).

**Table 3:** Comparison of patient safety culture dimensions between professional categories, Belo Horizonte, Minas Gerais, Brazil, 2021

Dimensions	Nursing	Medicine	Multidisciplinary	Administration
	PPR% (95%CI)^*^	PPR% (95%CI)^*^	PPR% (95%CI)^*^	PPR% (95%CI)^*^
Percentage of positive responses	55.8^†^ (61.2-50.4)	67.8^†^ (73.1-62.6)	61.3^†^ (67.3-55.4)	66.5^†^ (73.1-60.0)
D1 - Frequency of event reporting	48.3^‡^ (55.2-41.5)	63.8^†^ (66.8-60.7)	62.7^†^ (75.4-49.9)	79.3^§^ (89.3-69.3)
D2 - Perception of safety	53.2^†^ (86.9-53.2)	62.0^†^ (95.2-28.8)	46.8^‡^ (77.5-16.1)	65.2^†^ (86.3-44.2)
D3 - Supervisor/manager expectations & actions promoting safety	74.4^†^ (82.7-67.6)	78.8^§^ (84.1-73.6)	75.0^§^ (91.3-58.6)	77.4^§^ (85.7-69.0)
D4 - Organizational learning-continuous improvement	85.0^§^ (94.7-74.8)	87.3^§^ (105.3-69.3)	85.1^§^ (99.7-70.6)	100.0^§^ (100.0-100.0)
D5 - Teamwork within units	68.6^†^ (75.2-63.6)	88.6^§^ (93.1-84.1)	83.3^§^ (89.6-77.0)	63.8^†^ (74.3-53.4)
D6 - Communication openness	55.2^†^ (78.2-35.7)	62.0^†^ (83.7-40.3)	64.3^†^ (75.5-53.2)	50.0^†^ (81.4-18.5)
D7 - Feedback and communication about error	58.7^†^ (61.2-53.3)	61.5^†^ (72.5-50.5)	61.1^†^ (72.0-50.2)	76.8^§^ (89.5-64.1)
D8 - Nonpunitive response to error	26.3^‡^ (46.4-8.2)	40.6^‡^ (69.1-12.0)	35.1^‡^ (55.1-15.2)	35.4^‡^ (44.6-26.2)
D9 - Staffing	46.5^‡^ (71.1-25.5)	52.5^†^ (74.1-31.1)	47.2^‡^ (65.8-28.6)	44.8^‡^ (54.3-35.3)
D10 - Hospital management support for patient safety	60.1^†^ (67.4-53.9)	73.8^†^ (76.1-71.5)	63.4^†^ (79.1-47.6)	73.2^†^ (80.5-65.9)
D11 - Teamwork within units	53.5^†^ (63.3-44.2)	62.8^†^ (68.8-56.8)	55.5^†^ (64.4-46.6)	64.7^†^ (85.2-44.2)
D12 - Hospital handoffs & transitions	55.5^†^ (66.9-45.9)	57.8^†^ (70.7-44.9)	55.5^†^ (64.4-46.6)	63.9^†^ (78.1-49.7)

PRP - Porcentagem de Respostas Positivas; ^^*^^IC95% - Intervalo de Confiança de 95%;

† Dimensões classificadas como oportunidade de melhoria;

‡ Dimensões classificadas como fragilidades;

§ Dimensões classificadas como fortalezas.

When modeling the percentage of positive responses, it was identified in unadjusted analysis that only the type of management (x^2^=58.06; p<0.001) was related to the outcome. The private service (β=42.62) and the philanthropic service (β=43.24) show a greater trend of positive responses than the public service, with approximately more than 40 points (Table 4). Professional teams, working time in the hospital, working time in the unit, weekly working hours and working time in the specialty did not reveal minimal association in the unadjusted analysis (p>0.05).

**Table 4:** Unadjusted model for explaining the outcome percentage of total positive responses regarding patient safety culture in the work environment in dialysis services, Belo Horizonte, Minas Gerais, Brazil, 2021

Variables	unadjusted β	Wald 95%CI		Hypothesis test		
		Lower	Upper	x^2^ Wald	df	*p* value
Interception						
Service						
Private service	42.62	32.001	54.481	56.851	1	<0.001
Philanthropic service	43.24	25.400	52.192	32.221	1	<0.001
Public service	0					

*Unadjusted β _-_ Slope coefficient of patients’ overall safety score variable as the independent variable function; CI - Confidence Interval; df - degrees of freedom.*

## DISCUSSION

This study originally contributes to understanding the configuration of patient safety culture in the context of dialysis services and their predisposing factors, especially in the current pandemic scenario. In Brazil, no studies have yet been released that include the analysis of factors associated with patient safety culture in dialysis services and their comparison in relation to the different types of management of them.

There were differences between the perceptions of professionals in the different dialysis services, evidenced by the safety note and the areas of weakness/strengths of patient safety. It is believed that these differences are related to the degree of development of safety culture in the institution as well as the degree of freedom of expression of professionals in the services studied. Among the profile of the institutions studied, the public dialysis service is inserted in a hospital context, being a reference for the entire state in high complexity care and in treatment of COVID-19. In addition to meeting the outpatient demand, this service receives patients with hospitalization profile and greater severity potential, compared to other services in the network. During the pandemic, this institution absorbed a large number of patients who required dialysis, either due to the increased rate of acute kidney injury in patients affected by the severe form of SARS-CoV-2 in Intensive Care Units (ICU) or by the high number of patients with advanced chronic kidney disease. Such particularities may have contributed to the perception of worker safety from the public service^(6)^.

The areas for priority actions aimed at improving safety culture in services studied should focus on “D8” and “D9”, as these are the areas in which the lowest percentages of positive responses occurred, when the three services are analyzed together. At the same time, “D4” stands out, which, compared to the other dimensions, received the best assessment in the three services studied, and can be considered a strong area for safety culture. This perception shows that dialysis service professionals recognize that, in these institutions, there is a philosophy of continuous improvement and development strategies that favor professional improvement. These data are in line with a study conducted in six outpatient dialysis clinics in the countryside of New York, United States of America (USA), which obtained 80% of positive responses for this dimension^(21)^.

“D8” was the one that reached the lowest overall percentage in the three services studied, indicating that, for most professionals, errors that occurred and reported can be used against them and contribute to inhibiting behavior, discouraging reporting failures and adverse events^(22-23)^. This finding is similar to that of a study carried out at a university hospital in the capital of the Middle East, in which this dimension was considered an opportunity for improvement, as it had a low rate of positive response (26.8%)^(24)^. Complementing these data, a study conducted in southern Brazil^(25)^ pointed out that this dimension obtained one of the lowest scores of positive responses from the perspective of a health team of a university hospital ICU, a sector also considered critical and complex care, such as dialysis services. The weakness in this perception suggests the need to broaden the discussions on the theme about the culture of non-blame in health institutions, especially in public management, which obtained a worse assessment so that professionals have the freedom to express themselves and learn from mistakes without feeling at risk of punishment.

The weakness in “D9”, evidenced by the study, may reflect dissatisfaction with working conditions, excessive workload, exhausting working hours and work under pressure, having been observed with worse assessment also for public service professionals. Regarding this finding, a Brazilian study conducted in a public hospital in strength, Ceará, pointed out that staffing and workload are one of the main factors involved in patient safety culture^(26)^.

It is noteworthy that this assessment occurred in a critical period of the Brazilian health systems, in which there was a higher demand for service due to an exponential increase in the number of cases and hospitalizations by COVID-19, with a significant reduction in the workforce due to the absence of professionals due to illness or a situation of greater vulnerability, with significant staff impairment^(16)^. Although this overview was common to all hospital services, it is understood that, due to particularities of type of management in public services, especially those working under a public tender system, the process of replacing the staff may take longer than in other institutions. In this logic, maintaining an adequate size of professionals in hospital services, combined with safe care, has been a challenge.

The public service obtained a low percentage of positive responses in “D1”, “D2” and “D6”. In a study that also used the HSOPSC scale, carried out with nursing professionals in a large public general hospital, Bahia, Brazil, these dimensions were also among the areas of weaknesses. This fact reinforces the role of management in the face of strengthening actions and strategies aimed at improving quality of care, associated with control and monitoring mechanisms, commitment, transparency and collective responsibility, in order to achieve a better perception of patient safety^(27)^.

Regarding the distribution of positive responses, the privately management dialysis service was the one with the best results. Similarly, in a Peruvian study conducted in three teaching hospitals with different types of administration, a positive perception was obtained in seven dimensions and higher response scores in relation to the public sector^(28)^. This difference can be explained by the greater investment by managers of private institutions in coordinated, integrated and cooperative actions, in favor of improvement of work processes and the continuous search for quality, combined with the desire to remain in a competitive market, culminating in more favorable attitudes of professionals from the private sector for the collective construction of a patient safety culture^(29)^.

The stratified assessment of patient safety culture dimensions among the professional categories showed distinctions in the identification of strengths and weaknesses, in which physicians, multidisciplinary and administrative teams identified more areas of strength compared to the nursing team. Corroborating the findings of this research, gaps between perceptions in professional categories were also reported in an American study carried out with a multidisciplinary team in hemodialysis clinics, in which lower scores were observed for technical professionals and nurses, compared to other functions that included physicians, administrative leaders, nutritionists and social assistant^(30)^.

Another study carried through with professionals of Spanish hospitals showed that the position was a factor associated with safety culture assessment; however, this time, the nursing team presented the best positive perception^(31)^. It is believed that the distinction in patient safety culture perception and the ability to identify weaknesses may have been influenced by the degree of proximity to patients, since the study has results that, for the most part, reflect the perception of a class that spends most of the time with patients and that represents the largest category of professionals in the context of health services^(32)^. In this sense, nursing professionals would be more attentive in identifying risk situations to patient safety.

As in the analysis of services, “D4” was considered a strength for all professional categories, reinforcing that, in dialysis services, professionals recognize the existence of a culture that favors and makes possible improvement and personal development, fundamental requirements for good care practice.

In the assessment of possible influences of work and institutional factors on patient safety culture, a relationship was identified only for the type of management of dialysis services, with emphasis on the public service, pointed out as the weakest. A similar study, carried out in three hospitals in Brazil, also found significant associations between the type of hospital management and the overall patient safety score^(13)^. Thus, it is inferred that interventions to improve patient safety in environments of nephrological practices are planned considering the context of the type of administrative management, with emphasis on greater investments in public services, according to data from this research.

The triad composed of professional team, equipment and the environment can be considered a vulnerability factor for occurrence of adverse events in hemodialysis, recognized as a highly specific sector^(7)^. The path to improving and maintaining a strengthened safety culture in these sectors requires continuous assessments, based on personal development and investments in continuing education. Successful experiences were demonstrated by a Spanish study conducted in hospital hemodialysis units, which conducted assessment using the HSOPSC in two moments. There was a significant improvement in perception in seven safety culture dimensions, after implementation of training programs and implementation of adverse event reporting system^(33)^.

### Study limitations

The present study presented as a limitation the reduced sample size, characteristic of the number of professionals of dialysis services in a single municipality. Although the research had the participation of most professionals from the three services, the external validity of this research is limited. Characteristics may vary between regions of the country, cities or institutions of the same type of management. Another limiting factor is the use of an instrument that does not include the specificities of dialysis services, since the HSOPSC is more commonly used to assess the hospital environment in general.

### Contributions to nursing

This research provided a broad patient safety culture analysis in dialysis services from different administrative spheres in the pandemic context. It is believed that the results of this study may contribute to a better foundation on the subject, in addition to sensitizing professionals and managers about the importance of strategic actions for the continuous improvement of safety culture. It is expected that in each service, with different types of management, the results will be subsidies in the implementation and development of a strengthened culture in that service, in favor of preventing and mitigating errors and, consequently, improving health care quality.

## CONCLUSIONS

Patient safety culture analysis, according to the multidisciplinary team’s perception in the three hemodialysis services scenario of this study, with different types of management, found that “D4” can be considered a strengthened area and that there are variations of views between professional categories. Although most professionals classify safety as good, strengths in the safety culture dimensions were noticeable in two dimensions, “D3” and “D4”, and were more concentrated in private and philanthropic service. The study allowed the identification of weaknesses, namely “D8” and “D9”, which should receive attention and priority in the planning of interventions for improvement, especially in the public service, where there was a concentration of greater weak areas to be worked on. Interventions should consider the type of service management, as it is an intervening factor in the general perception of professionals. Furthermore, the importance of top management in the implementation processes and the development of a safety culture focused on continuous improvement of quality of care in hemodialysis services is highlighted. It is suggested that the present study be reproduced in order to better understand possible gaps in safety culture perception that may have been influenced by the context of the COVID-19 pandemic.

## SUPPLEMENTARY MATERIAL

https://doi.org/10.48331/scielodata.GOVQBC
